# Effects of Ferri Ferrocyanas in Chorea

**Published:** 1840-05-16

**Authors:** William Zollickoffer

**Affiliations:** Middleburg, (Md.)


*Middleburg, (Md.) 7th May*, 1840.

### EFFECTS OF FERRI FERROCYANAS IN CHOREA.

BY WILLIAM ZOLLICKOFFER, M. D.

To the Editors of the Medical Examiner.

GENTLEMEN,—In December, 1839, the daughter of Mr. Haugh, twelve years old, claimed my professional attention. She had been labouring under chorea about four weeks before Mr. H. called on me to visit her. At the time I commenced treating her malady, it had assumed rather an aggravated form. Supposing that the disease was attributable to gastro-intestinal irritation from worms, I directed successively the various anthelmintics in use; which were succeeded by the exhibition of castor oil, senna, and salts, without procuring the dislodgement of even a single worm, or mitigating the violence of the paroxysm. To carry out this plan of treatment required ten days, during which period I procured frequent and copious dejections. I now concluded that the disease did not arise from irritation of the stomach and bowels, occasioned from worms, the accumulation of fecal matter, or any irritating substance located in the intestinal canal. That it did not exist as a consequence of suppressed catamenia was very obvious, as the child had not arrived at the period at which the menses usually makes its appearance.

Influenced by these views, I deemed it advisable to direct the use of such remedial agents as were calculated to produce an impression on the general system. I commenced this course with camphor, in association with opium, without any benefit. I then substituted quinine, in combination with assafœtida, and the result was the same. The cuprum ammoniacum was then given a fair trial; and the nitras argenti, and subcarbonas ferri præparatus, successively, without the least visible alteration in arresting the violence of the disease. Last of all, I concluded, pretty much as a dernier resort, to give the ferri ferrocyanas a fair trial, and commenced with doses of three grains, made into pills, three times a day; and, in six days, the malady was entirely removed, and the child has been perfectly free from the slightest symptom or evidence of the disease ever since. This is the second case in which I have experienced the good effects in chorea from the use of the prussiate of iron.

